# Effects of Resveratrol in Pregnancy Using Murine Models with Reduced Blood Supply to the Uterus

**DOI:** 10.1371/journal.pone.0064401

**Published:** 2013-05-08

**Authors:** Rajan Poudel, Joanna L. Stanley, Christian F. Rueda-Clausen, Irene J. Andersson, Colin P. Sibley, Sandra T. Davidge, Philip N. Baker

**Affiliations:** 1 University of Alberta, Edmonton, Alberta, Canada; 2 Maternal and Fetal Health Research Centre, University of Manchester and Central Manchester University Hospitals NHS Foundation Trust, United Kingdom; 3 Women and Children's Health Research Institute, Edmonton, Alberta, Canada; 4 Liggins Institute, University of Auckland, New Zealand; VU University Medical Center, The Netherlands

## Abstract

Preeclampsia (PE) and fetal growth restriction (FGR) contribute significantly to fetal and maternal morbidity and mortality. Although the causes of PE and FGR are not fully understood, both conditions are known to be associated with impaired uterine artery blood flow. Resveratrol, a polyphenol found in a number of plants, has been shown to induce relaxation of uterine arteries *in vitro* as well as improve many pathological conditions associated with PE and FGR. We hypothesized that treatment of endothelial nitric oxide synthase knockout mice (eNOS^−/−^) and catechol-O-methyltransferase knockout mice (COMT^−/−^) with resveratrol during pregnancy would improve uterine artery blood flow and therefore ameliorate the PE-like phenotype and FGR in these murine models. Pregnant C57BL/6J, eNOS^−/−^ and COMT^−/−^ mice received either resveratrol supplemented diet (4 g/kg diet) or control diet between gestational day (GD) 0.5 and GD 18.5. Resveratrol supplementation significantly increased uterine artery blood flow velocity and fetal weight in COMT^−/−^ but not in eNOS^−/−^ mice. There were no effects of resveratrol on litter size and placental weight among the groups. In conclusion, resveratrol increased uterine artery blood flow velocity and fetal weight in COMT^−/−^ mice, suggesting potential as a therapeutic strategy for PE and FGR.

## Introduction

Preeclampsia (PE) and fetal growth restriction (FGR) complicate over 10% of all human pregnancies and contribute significantly to fetal and maternal morbidity and mortality [1]. PE is defined as high blood pressure and proteinuria after the 20^th^ week of gestation [2]. FGR is defined as a fetus that fails to reach its genetic growth potential [3]. Long-term effects of PE and small size at birth include neurological and developmental delay and an increased risk of developing cardiovascular disease and diabetes in adult life [4], [5]. At present the only curative treatment for PE is the delivery of the placenta.

The etiologies of PE and FGR are complex and not fully understood, but both conditions are associated with impaired uterine artery blood flow [6], [7]. PE and FGR are associated with decreased trophoblast invasion of the maternal spiral arteries, which leads to increased resistance and therefore impaired uterine artery blood flow [8], [9]. In FGR, impaired uterine artery flow can result in diminished oxygen and nutrient delivery to the fetus [10]. In PE, this leads to the release of circulating factors, culminating in widespread acute maternal endothelial dysfunction and multi-organ failure [11]. Thus, treatments directed at improving uterine artery blood flow have a theoretical potential as therapies to ameliorate PE and FGR.

We, and others, have used mice deficient in endothelial nitric oxide synthase (eNOS^−/−^) and mice lacking Catechol-O-methyl transferase (COMT^−/−^), both of which share many phenotypic characteristics of PE and FGR [12], [13], [14], [15], [16]. During pregnancy, eNOS^−/−^ mice exhibit maternal features of PE including hypertension [14] and increased placental oxidative stress [15]. Moreover, these mice deliver growth-restricted fetuses [17], [16] and exhibit reduced uterine artery diameter and uterine artery blood flow [16]. Pregnant COMT^−/−^ mice exhibit many features of PE including raised blood pressure, proteinuria and placental abnormalities [12] and FGR [13]. Furthermore, uterine arteries from pregnant COMT^−/−^ mice *ex vivo* exhibit an increased constriction in response to phenylephrine (Phe) compared to arteries from control mice [13].

Resveratrol (3,5,4′-trihydroxystilbene) is a naturally occurring polyphenol [18] that could have beneficial effects by targeting some of the common pathophysiological mechanisms described in PE and FGR. The beneficial effects of resveratrol appear to be mediated via a plethora of pathways including enhanced NO bioavailability through endothelial NO synthase expression, as well as a reduction in oxidative stress, improvement of mitochondrial oxidative capacity and a decrease in ischemia reperfusion injury [19].

Previous studies have evaluated safety, pharmacokinetics and metabolism of resveratrol and have reported resveratrol to be well tolerated in humans even at very high doses [20] and no evidence of teratogenesis associated with this compound was found in rodents [21]. Although previous use of resveratrol in animal models of PE and is limited, [22] it has been shown to ameliorate high blood pressure, [23] proteinuria [24] and improve fetal weight [25]. In addition, resveratrol induces vasorelaxation of uterine arteries in non-pregnant guinea pigs [26].

We tested the hypothesis that supplementing the diet of eNOS^−/−^ and COMT^−/−^ mice with resveratrol during pregnancy will improve uterine artery blood flow velocity and therefore ameliorate the PE like phenotype and FGR in these murine models.

## Materials and Methods

Experiments were approved by the University of Alberta in accordance with the Canadian Council on Animal Care guidelines.

### Animals

Female COMT^−/−^ (n = 16) (Courtesy of Professor J Gogos, Columbia University), eNOS^−/−^ (n = 21) and C57BL/6J mice (n = 14) (Jackson Laboratories; Bar Harbor, ME) of 2–3 months of age were mated with strain-matched males nightly. The day of vaginal plug detection was designated as gestational day (GD) 0.5. Dams were randomly assigned to receive purified control diet (AIN-93G diet, Dyets Inc., Bethlehem, PA) or AIN-93G diet supplemented with 4-gram resveratrol/kg diet from gestational day (GD) 0.5 to GD 18.5. The duration of administration and dose of resveratrol in the diet was based on a previous study [27]. In addition, a therapeutic concentration range of resveratrol in the plasma was achieved following the dose administered [28].

### Blood pressure and proteinuria

Mice were trained in restraint tubes for 5 minutes each on three successive days prior to mating. Blood pressure was measured using tail-cuff plethysmography (IITC Life Science, CA, USA) on GD 17.5 as previously described [13]. The method utilized is known to measure systolic blood pressure (SBP) accurately. A software algorithm calculates diastolic blood pressure (DBP); therefore, it is only estimation rather than a true measurement.

Urine was collected on GD 18.5 of pregnancy and stored at −80**°**C. Urine albumin (AssayPro, MO, USA) and creatinine (Cayman Chemical Company, MI, USA) kits were used to measure respective concentrations.

### Ultrasound biomicroscopy

Uterine and umbilical artery blood flow velocity was assessed *in vivo* under anesthesia on GD 17.5 using an ultrasound biomicroscope (model Vevo 2100, VisualSonics**®**, ON, Canada) as described previously [29], [13]. Briefly, uterine artery Doppler waveforms were obtained from both left and right uterine artery, and the umbilical artery from at least two fetuses were assessed. Peak systolic velocity (PSV) and end diastolic velocity (EDV) were measured at from three cardiac cycles and the results were averaged.

### Fetal and placental measurements

Dams were euthanized on GD 18.5 and the entire uterus was removed. Pups and placentas were dissected out and litter size was recorded. In addition, all fetuses were examined for external malformations. The fetuses and placentas were blotted dry and weighed; fetal crown to rump length and abdominal circumference were recorded.

### Assessment of uterine artery function ex vivo

Uterine arteries from the right horn were carefully dissected in cold PSS and surrounding adipose and connective tissue was removed. They were then cut into four small segments and mounted on a wire myograph (610 m, Danish Myo Technology, Aarhus, Denmark) as described elsewhere [13]. Vessel function was then assessed as described previously [13] by measuring contraction response to Phe, endothelium-dependent relaxation response to methacholine (Mch) and endothelium-independent relaxation to the NO-donor sodium nitroprusside (SNP).

### Statistical analysis

All data is presented as mean ± standard error of the mean (SEM) and compared using two-way ANOVA that included genotype and resveratrol treatment as independent variables; significance was determined using Bonferroni post-hoc test. Statistical significance was defined as P<0.05. Graphpad Prism 5.0 software was used for statistical analysis. For wire myography experiments, sigmoidal dose response curve fittings were performed and EC50 concentration of the drug was calculated using Graphpad Prism 5.0.

## Results

### Blood pressure and proteinuria

Systolic blood pressure was not significantly different among the groups and there was no effect of resveratrol on this measure. A decrease in SBP was observed in the eNOS^−/−^ mice following resveratrol treatment, however it did not reach statistical significance (Table 1). There was no difference in DBP among the groups and resveratrol did not have any effect on this parameter (Table 1).

**Table 1:** Effect of resveratrol on maternal blood pressure on GD 17.5 in C57BL/6J, eNOS^−/−^ and COMT^−/−^ mice.

	SBP (mmHg)	DBP (mmHg)
**C57BL6J + CD (n = 7)**	133±2	98±6
**C57BL6J + RD (n = 7)**	134±4	85±8
**eNOS** ^−/−^ **+ CD (n = 11)**	131±4	87±3
**eNOS** ^−/−^ **+ RD (n = 10)**	118±3	75±4
**COMT** ^−/−^ **+ CD (n = 6)**	131±7	89±8
**COMT** ^−/−^ **+ RD (n = 9)**	138±5	96±5

CD: Control diet, RD: Resveratrol diet, SBP: Systolic blood pressure, DBP: Diastolic blood pressure. Mean ± SEM, two-way ANOVA.

**Figure 1:**
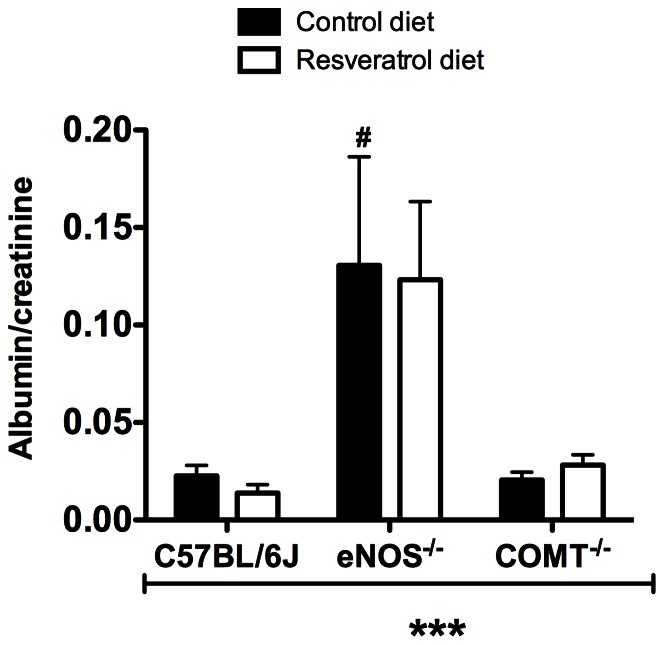
Effect of genotype and resveratrol treatment on proteinuria. There was a significant effect of genotype on Albumin:creatinine ratio. This was significantly elevated in the eNOS^−/−^ mice. There was however no effect of resveratrol treatment in this measure. Mean ± SEM, n = 5–7, ***p<0.001 two-way ANOVA, #p<0.05, Bonferroni post-hoc test indicating differences within genotypes in control diet.

There was a significant effect of genotype on maternal proteinuria at GD 18.5 (Figure 1; p<0.001). A significantly higher albumin: creatinine ratio was observed in eNOS^−/−^ compared to C57BL/6J on control diet. Supplementation with resveratrol, however, had no effect on proteinuria.

### Uterine and umbilical artery blood flow velocity

**Figure 2:**
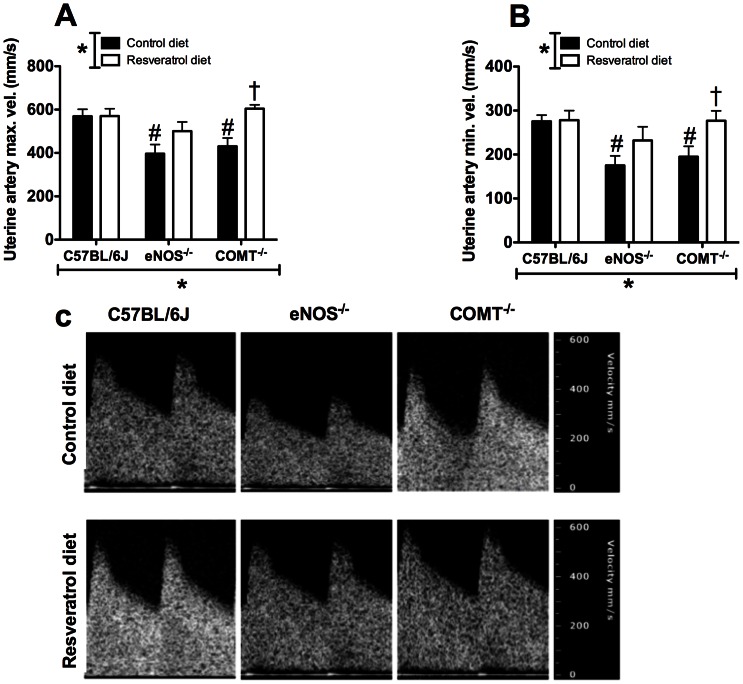
Effect of genotype and resveratrol treatment in uterine artery Doppler indices. There was a significant effect of genotype on both maximum (A) and minimum (B) uterine artery blood flow velocity. Resveratrol treatment significantly increased both maximum and minimum uterine artery blood flow velocity in COMT^−/−^ mice. A significant interaction of genotype and treatment on maximum uterine artery blood flow velocity (p<0.05, two-way ANOVA) was observed. (C) Example uterine artery Doppler waveforms from C57BL6/J, eNOS^−/−^ and COMT^−/−^ mice ± Resveratrol. Mean ± SEM, n = 6-10, *p<0.05, two-way ANOVA, #p<0.05, Bonferroni post-hoc test indicating differences within genotypes in control diet. †p<0.05 Bonferroni post-hoc test indicating differences between control and resveratrol diet within the same genotype.

A significant effect of genotype on both maximum (Figure 2A) and minimum (Figure 2B) uterine artery blood flow velocity was observed (p<0.05). In addition, there was a significant interaction of genotype and treatment on maximum uterine artery blood flow velocity (p<0.05). Interestingly, resveratrol treatment increased both of these measures in COMT^−/−^ mice but not in C57BL/6J; in eNOS^−/−^ mice trends to increased blood flow velocity were not significant (Figure 2). There were no effects of genotype or resveratrol treatment on umbilical artery blood flow velocity in either of the groups.

A complete assessment of uterine and umbilical artery hemodynamic and waveforms parameters are listed in Table S1.

### Maternal weight gain and litter size

Maternal weight gain during pregnancy was not different among the groups and there was no effect of resveratrol treatment on this measure (C57BL6/J; 11.9±0.6 vs 12.0±1.2, eNOS^−/−^; 10.9±0.1 vs 10.41±0.6, COMT^−/−^ 12.9±0.4 vs 12.0±1.01 g).

Additionally, there was no effect of genotype or resveratrol treatment on litter size in either of the groups (C57BL6/J; 6.5±0.3 vs 7.2±0.6, eNOS^−/−^; 7.1±0.8 vs 6.6±0.5, COMT^−/−^ 7.8±0.3 vs 7.8±0.9).

### Fetal and placental measurements

**Figure 3:**
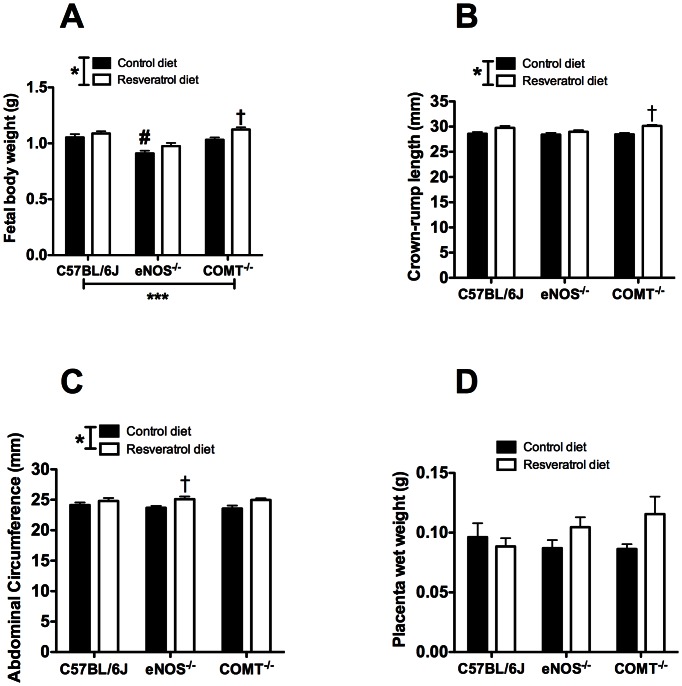
Effect of the genotype and resveratrol treatment in pup weight. A) Pup weight was significantly affected by both genotype and treatment. A significant reduction in pup weight in eNOS^−/−^ mice was observed. There was a significant increase in pup weight in COMT^−/−^ mice following resveratrol administration. B) There was no effect of genotype or resveratrol treatment on placental weight for all groups. C) Resveratrol treatment leads to significant increase in crown-rump length only in the COMT^−/−^ group. D) Resveratrol treatment leads to significant increase in abdominal circumference in eNOS^−/−^ mice. Mean ± SEM, n = 7–11, ***p<0.001, *p<0.05, two-way ANOVA, #p<0.05, Bonferroni post-hoc test indicating differences within genotypes in control diet. †p<0.05 Bonferroni post-hoc test indicating differences between control and resveratrol diet within same genotype.

There was a significant effect of both genotype and treatment on pup weight (p<0.001, Figure 3A). Pup weight in untreated eNOS^−/−^ mice was significantly reduced compared to C57Bl/6J mice on control diet. There was a trend towards an increase in pup weight in resveratrol treated eNOS^−/−^ mice (0.910±0.02 vs 0.975±0.02 g), although the difference did not reach statistical significance at the 5% level. A significant increase in pup weight was observed in COMT^−/−^ (1.031±0.02 vs 1.119±0.01 g, p<0.05; Figure 3A) but not in C57BL/6J (1.052±0.02 vs 1.088±0.01 g) mice following resveratrol administration. Crown to rump length was significantly higher in the resveratrol treated COMT^−/−^ (28.4±0.2 vs 30.14±0.2 mm; p<0.05; Figure 3B) but not in eNOS^−/−^ (28.3±0.3 vs 28.9±0.3 mm) or C57BL/6J (28.5±0.2 vs 29.7±0.3 mm) mice. Abdominal circumference was significantly larger in resveratrol treated eNOS^−/−^ compared with untreated genotype controls (23.6±0.3 vs 25.0±0.4 mm, p<0.05; Figure 3C) but not in COMT^−/−^ (23.5±0.5 vs 24.9±0.2) or C57BL/6J (24.1±0.4 vs 24.8±0.4 mm) mice. Placental weight was not affected by either genotype or resveratrol treatment (Figure 3D).

### Uterine artery ex vivo vascular function

**Figure 4:**
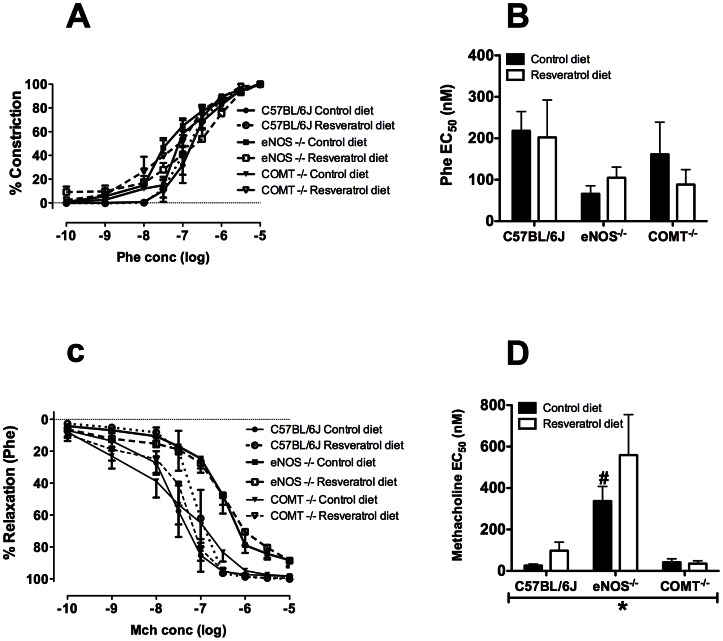
Effect of genotype and resveratrol treatment on sensitivity to phenylephrine and methacholine. A) Phe induced vasoconstriction of the uterine arteries in C57BL/6J, eNOS^−/−^ and COMT^−/−^ mice. B) There was no effect of genotype or treatment on phenylephrine EC_50_ concentration. C) Percent relaxation induced by methacholine in C57BL/6J, eNOS^−/−^ and COMT^−/−^ mice. D) There was a significant effect of genotype on the methacholine EC_50_ concentration such that it was significantly increased in eNOS^−/−^ mice. Mean ± SEM, n = 5–6, ***p<0.001, two-way ANOVA, #p<0.05, Bonferroni post-hoc test indicating differences within genotypes in control diet.

Sigmoid curves in response to Phe-induced constriction are presented on Figure 4A. Sensitivity to Phe was not different in any of the groups (Figure 4B). Sigmoid curves in response to Mch-induced relaxation are presented on Figure (Figure 4C). Sensitivity to Mch was significantly impaired in eNOS^−/−^ compared with C57BL/6J mice (Figure 4C). Supplementation with resveratrol did not improve this impairment (Figure 4D). No difference in uterine artery relaxation was observed among the groups in response to the NO donor SNP (data not shown).

## Discussion

Despite intensive research and clinical trials there are currently no therapeutic approaches available for either treatment or prevention of PE and FGR. Although maternal manifestations differ, both of these conditions are associated with impaired uterine artery blood flow. In the present study, resveratrol administration during pregnancy led to significant increases in uterine artery blood flow velocity and concomitant increases in fetal weight in COMT^−/−^ mice. In the eNOS^−/−^ mice, increases in uterine artery blood flow velocity and fetal weight following resveratrol administration did not reach significance.

Many clinical studies have shown a decrease in uteroplacental blood flow in cases of PE and FGR [30], [31]. In this study there was a significant effect of genotype on uterine artery blood flow velocity. Further, uterine artery blood flow velocity was significantly increased in the COMT^−/−^ mice following resveratrol administration. These results are dissimilar to a previous study [22] in a rat model of PE, which showed no difference in blood flow to placenta following resveratrol administration. The reason for these differences may be due to variation in timing and dosage of resveratrol in addition to methodological differences in measuring blood flow. There is some indirect evidence to support the uterine artery blood flow velocity increase observed in COMT^−/−^ mice receiving resveratrol. Resveratrol is known to increase cerebral blood flow in humans, [32] blood flow to the kidney in a mouse model of sepsis-induced acute kidney injury, [33] and in coronary arteries in a swine model of experimental acute coronary occlusion [34].

The mechanisms by which resveratrol induces increases in uterine artery blood flow velocity in COMT^−/−^ mice may be through vasorelaxation of uterine arteries. In fact, Naderali et al [26] demonstrated that resveratrol is able to induce significant vasorelaxation in uterine arteries from guinea pigs. In addition, pre-treatment of the uterine arteries with L-NAME (a NOS inhibitor) had no effect on resveratrol induced vasorelaxation, [26] suggesting that resveratrol induced vasorelaxation through NO independent mechanisms. When assessed *in vivo*, resveratrol has been demonstrated to increase both endothelial and inducible NO synthase [35]. Therefore, resveratrol could induce vasorelaxation of uterine arteries in COMT^−/−^ mice through multiple mechanisms. Interestingly, resveratrol treatment did show a trend to increase uterine artery blood flow velocity in the eNOS^−/−^ mice but this did not reach significance. It is plausible that in part, resveratrol increased uterine artery blood flow velocity in the COMT^−/−^ mice via upregulation of eNOS. However, this may not be likely as our *ex vivo* studies show no difference in endothelium dependent relaxation of uterine arteries in C57BL/6J and COMT^−/−^ groups. In agreement with our previous study [17], eNOS^−/−^ mice, however, exhibited significantly impaired endothelium dependent relaxation of uterine arteries. This impairment was not improved following resveratrol administration. Taken together these data indicate that resveratrol may increase uterine artery blood flow velocity in the COMT^−/−^ mice through NO independent pathway but have minimal effects on eNOS^−/−^ mice.

Following resveratrol treatment, pups from COMT^−/−^ mice showed a significant increase in pup weight, this may be attributed to the observed increase in uterine artery blood flow velocity. Our results are consistent with those of Sing et al, [25] who reported an increase in embryo weight and crown-rump length following resveratrol administration in a rat model of diabetic embryopathy, which, similar to PE and FGR, is associated with a decrease in embryo weight, an increase in oxidative stress and endothelial dysfunction.

Hypertension and proteinuria associated with PE pose a serious risk to maternal health and are linked to adverse neonatal outcomes. In this study there was no significant difference in systolic blood pressure between C57BL/6J, eNOS^−/−^ and COMT^−/−^ mice. The blood pressure results in COMT^−/−^ mice in the current study are consistent with our previous findings, [13] however, differ from those of Kanasaki and colleagues, [12] who observed a significant increase in systolic blood pressure in COMT^−/−^ mice at GD 17.5. In terms of the eNOS^−/−^ mice, there were no differences in systolic and diastolic blood pressure when compared to C57BL/6J mice. Hefler et al [14] reported a significant increase in blood pressure in eNOS^−/−^ mice during pregnancy, while Shesely et al [36] reported a decrease in blood pressure in eNOS^−/−^ mice during pregnancy. Consistent with the present study, all of the aforementioned studies used the tail-cuff method to measure blood pressure. The apparent discrepancy in blood pressure results across studies may be resolved by using telemetry, which is known to reduce variability and artifacts in blood pressure results associated with the tail-cuff method [37]. Although resveratrol has been shown to reduce blood pressure in other animals models of human diseases [23], [38] it did not have any effect on blood pressure in this study. This lack of efficacy may be due to the fact that COMT^−/−^ and eNOS^−/−^ mice were not hypertensive in the current study, unlike the animal models tested in other preclinical studies. Antihypertensive treatments used in women with PE often pose a dilemma among clinicians because of their risk of decreasing blood flow by reducing perfusion pressure to an already compromised uteroplacental unit [39]. A drug that is known to reduce maternal blood pressure and improve uteroplacental blood flow is currently unavailable. It is therefore necessary to explore the use of resveratrol in an animal model that exhibits hypertension during pregnancy, particularly since it increases uterine artery blood flow velocity in the COMT^−/−^ mice.

The increase in proteinuria observed in eNOS^−/−^ mice is in agreement with a study by Kusinski et al, [15] however, proteinuria results in COMT^−/−^ mice this study are dissimilar to two previous studies [12], [13]. The reason for absence of proteinuria in the current study may be explained by the lack of hypertension observed in the COMT^−/−^ mice.

In summary, resveratrol treatment leads to an increase in pup growth in COMT^−/−^ mice, which may be mediated by the increase in uterine artery blood flow velocity also found in this disease model. In addition, there was no evidence of treatment-related external malformations or effects on litter size in any of the mouse models tested. Although our study indicates that resveratrol may offer therapeutic potential in FGR and PE, further studies are needed to verify its potential in phenotypically stronger models of PE and FGR.

## Supporting Information

Information S1 Velocity-time integral of Doppler signal, *: p<0.05 when effect of genotype (Gen), administration of resveratrol (Resv) or their interaction (Int) was evaluated by two-way ANOVA. #p<0.05, Bonferroni post-hoc test indicating differences within genotypes in control diet.
(DOCX)
