# Supplementary material for: Effects of Resveratrol in Pregnancy Using Murine Models with Reduced Blood Supply to the Uterus
Source: PLoS One. 2013 May 8;8(5):e64401. doi: 10.1371/journal.pone.0064401 (PMC3648569; doi:10.1371/journal.pone.0064401)
Supplement: Information S1 — Velocity-time integral of Doppler signal, *: p<0.05 when effect of genotype (Gen), administration of resveratrol (Resv) or their interaction (Int) was evaluated by two-way ANOVA. #p<0.05, Bonferroni post-hoc test indicating differences within genotypes in control diet. (DOCX) [file pone.0064401.s001.docx]

Table S1: Hemodynamic parameters of uterine and umbilical vasculature evaluated by ultrasound biomicroscopy at gestational age 17.5

|  | **C57BL6/J** | | **eNOS-/-** | | **COMT-/-** | | **ANOVA** | | |
| --- | --- | --- | --- | --- | --- | --- | --- | --- | --- |
|  | **Control diet** | **Resv diet** | **Control diet** | **Resv diet** | **Control diet** | **Resv diet** | **Int** | **Gen** | **Resv** |
| Maternal Heart rate (bpm) | 585 ± 15 | 565 ± 14 | 608 ± 10 | 591 ± 14 | 588 ± 10 | 577 ± 11 |  |  |  |
| Avg. fetal heart rate (bpm) | 227 ± 7 | 229 ± 12 | 253 ± 7 | 229 ± 3 | 243 ± 10 | 249 ± 14 |  |  |  |
| **Uterine Artery** |  |  |  |  |  |  |  |  |  |
| VTI (cm) | 41 ± 2 | 41 ± 4 | 53 ± 19 | 85 ± 33 | 32 ± 2 | 51 ± 6 |  |  |  |
| Max vel (mm/s) | 569 ± 32 | 554.61 ± 36 | 396± 41 # | 500 ± 42 | 430 ± 35 # | 604 ± 17 | * | * | ** |
| Mean Vel (mm/s) | 411 ± 21 | 402 ± 27 | 255 ± 40 | 286 ± 39 | 299 ± 28 | 426 ± 18 |  | *** | * |
| Min vel (mm/s) | 275 ± 14 | 278 ± 21 | 175 ± 21# | 231 ± 31 | 195 ± 22 # | 276 ± 22 |  | * | * |
| Mean gradient (mmHg) | 0.59 ± 0.07 | 0.66 ± 0.08 | 0.29 ± 0.06 | 0.37 ± 0.08 | 0.38 ± 0.07 | 0.73 ± 0.06 | * | *** | * |
| **Umbilical Artery** |  |  |  |  |  |  |  |  |  |
| VTI (cm) | 14 ± 1 | 12 ± 0.9 | 12 ± 2 | 17 ± 2 | 11 ± 0.9 | 15 ± 2 |  |  |  |
| Max vel (mm/s) | 145 ± 11 | 119 ± 4 | 103 ± 11 | 129 ± 19 | 119 ± 7 | 150 ± 22 |  |  |  |
| Mean Vel (mm/s) | 71 ± 5 | 61 ± 3 | 49 ± 8 | 65 ± 9 | 59 ± 3 | 74 ± 10 |  |  |  |
| Min vel (mm/s) | 9 ± 0.9 | 10 ± 1 | 12 ± 1 | 9 ± 1 | 8 ± 1 | 13 ± 4 |  |  |  |
| Mean gradient (mmHg) | 0.021 ± 0.003 | 0.015 ± 0.001 | 0.012 ± 0.001 | 0.019 ± 0.006 | 0.015 ± 0.001 | 0.024 ± 0.007 |  |  |  |
